# Natural Antibodies Produced in Vaccinated Patients and COVID-19 Convalescents Hydrolyze Recombinant RBD and Nucleocapsid (N) Proteins

**DOI:** 10.3390/biomedicines12051007

**Published:** 2024-05-02

**Authors:** Anna M. Timofeeva, Liliya Sh. Shayakhmetova, Artem O. Nikitin, Tatyana A. Sedykh, Andrey L. Matveev, Daniil V. Shanshin, Ekaterina A. Volosnikova, Iuliia A. Merkuleva, Dmitriy N. Shcherbakov, Nina V. Tikunova, Sergey E. Sedykh, Georgy A. Nevinsky

**Affiliations:** 1 SB RAS Institute of Chemical Biology and Fundamental Medicine, 630090 Novosibirsk, Russiasedyh@niboch.nsc.ru (S.E.S.);; 2 Advanced Engineering School, Novosibirsk State University, 630090 Novosibirsk, Russia; 3 State Research Center of Virology and Biotechnology Vector, 630559 Koltsovo, Russiadnshcherbakov@gmail.com (D.N.S.); 4 Department of Physical-Chemistry, Biology and Biotechnology, Altay State University, 656049 Barnaul, Russia

**Keywords:** SARS-CoV-2, COVID-19, catalytic antibodies, IgG, autoimmunity, N-protein, RBD, coronavirus, proteolytic antibodies

## Abstract

Antibodies are protein molecules whose primary function is to recognize antigens. However, recent studies have demonstrated their ability to hydrolyze specific substrates, such as proteins, oligopeptides, and nucleic acids. In 2023, two separate teams of researchers demonstrated the proteolytic activity of natural plasma antibodies from COVID-19 convalescents. These antibodies were found to hydrolyze the S-protein and corresponding oligopeptides. Our study shows that for antibodies with affinity to recombinant structural proteins of the SARS-CoV-2: S-protein, its fragment RBD and N-protein can only hydrolyze the corresponding protein substrates and are not cross-reactive. By using strict criteria, we have confirmed that this proteolytic activity is an intrinsic property of antibodies and is not caused by impurities co-eluting with them. This discovery suggests that natural proteolytic antibodies that hydrolyze proteins of the SARS-CoV-2 virus may have a positive impact on disease pathogenesis. It is also possible for these antibodies to work in combination with other antibodies that bind specific epitopes to enhance the process of virus neutralization.

## 1. Introduction

Catalytic antibodies possess the ability to bind antigens and catalyze specific reactions, similar to the function of enzymes [1,2,3]. The first report on natural proteolytic antibodies dates back to 1989 [4,5]. The peptides were obtained from the blood plasma of patients with bronchial asthma and were observed to hydrolyze vasoactive intestinal peptides (VIP) [4]. That study provided the first indication that antibodies possessing catalytic activity can be produced in vivo without immunization with an artificial hapten. Recent findings have reported the presence of natural catalytic antibodies in patients with various pathologies, including viral and autoimmune diseases [5,6,7,8,9,10]. For example, patients with thyroiditis, multiple myeloma, and hemophilia have been found to have catalytic antibodies against thyroglobulin, prothrombin, and factor VIII, respectively [11,12,13,14]. Antibodies associated with multiple sclerosis (MS) [15] and systemic lupus erythematosus (SLE) enzymatically degrade myelin basic protein (MBP) [16]. Amyloid β peptide (Aβ)-hydrolyzing IgM antibodies were recently found in the sera of patients with Alzheimer’s disease (AD) [17,18,19,20]. IgG and IgM from HIV-infected patients hydrolyze HIV reverse transcriptase, integrase [21], and the envelope proteins gp120 [10,22,23,24,25] and gp4 [26].

Many reports have been published concerning the COVID-19 pandemic and the SARS-CoV-2 virus. However, only a few studies have reported catalytically active antibodies in patients after COVID-19. Our investigation in 2023 revealed that antibodies obtained from COVID-19 convalescents and vaccinated donors can hydrolyze six out of nine oligopeptides that correspond to epitopes of the S-protein of SARS-CoV-2 [27]. We also identified distinct hydrolysis sites for proteolytic antibodies [28] and demonstrated the proteolytic activity of plasma antibodies from COVID-19 convalescents for the hydrolysis of an S-protein and two oligopeptides corresponding to the RBD epitopes of SARS-CoV-2 virus [29].

This article reports on a comprehensive characterization of the proteolytic activity of antibodies targeting two proteins of the SARS-CoV-2 virus: the N-protein of the nucleocapsid and the S-protein and its fragment RBD located on the virion surface. We compared the ability of antibodies against S-protein, RBD, and N-protein in COVID-19 convalescents and vaccinated with Sputnik V (based on Ad26 and Ad5 adenoviruses) to hydrolyze the corresponding substrates. The article outlines the criteria for establishing the association of catalytic activity with antibodies developed by the research teams of S. Paul and G.A. Nevinsky. Additionally, a description of the individual features of catalytic antibodies in individual patients is provided.

## 2. Results

### 2.1. Characterization of Donors and Isolation of Antibodies and Their Subfractions

COVID-19 convalescents have been observed to generate antibodies to various viral proteins. However, vaccination with the adenovirus-based Sputnik V vaccine resulted in the production of antibodies only to the S-protein of SARS-CoV-2 but not to other viral proteins [30]. Antibodies against the S-protein, RBD, and N-proteins of the SARS-CoV-2 virus in donor plasma were determined using ELISA. The assay allowed for the classification of donors into groups, with asymptomatic donors excluded from the vaccinated and disease-free donor groups.

The questionnaire data and ELISA results were analyzed to classify the donors into four groups, with 20 individuals in each group. These groups include Con+Vac (COVID-19 convalescents subsequently vaccinated with Sputnik V), Con (COVID-19 convalescents), Vac (those vaccinated with Sputnik V and who did not have COVD-19), and Neg (those who did not have COVID-19 and were not vaccinated).

Convalescent COVID-19 donors and vaccinated donors with the highest titers to S-protein were selected for antibody analysis. The groups were equalized by sex and age. Only patients and donors with no history of autoimmune pathologies and chronic infections were included in the study. Polyclonal IgG preparations from blood plasma were isolated via affinity chromatography on Protein-G-Sepharose.

Unfortunately, the number of antibodies demonstrating affinity to viral proteins within the total antibody pool was insufficient for individual isolation, as previously indicated [31]. The content of the RBD-IgG reached a maximum of 1.5% of all IgG preparations, while the N-IgG content reached a maximum of 0.5%. Therefore, the IgG preparations specific to viral proteins (S-protein, N-proteins, and RBD—the domain within the S-protein) were obtained from the total equimolar antibody preparation of patients in each group. Antibody fractionation was performed via affinity chromatography on RBD-, S-, and N-Sepharoses.

The IgG preparations were isolated for each donor, and antibody fractions with affinity to the RBD, S-protein, and N-proteins were obtained for three groups of donors (Con+Vac, Con, and Vac): RBD-IgG, S-IgG, and N-IgG, respectively. The chromatography profiles were similar to those obtained previously [31] and are not reported in this work. The IgG preparation of blood plasma from donors without antibodies to the SARS-CoV-2 virus was used as a control.

### 2.2. Analysis of the Catalytic Activity of Antibodies in the Hydrolysis of RBD and N-Protein of the SARS-CoV-2 Coronavirus

Extensive glycosylation of the S-protein covers approximately 40% of the protein surface, sterically protecting many epitopes from immune recognition [32]. The RBD is a region of the S-protein, and since the RBD is largely devoid of glycosylation and contains important residues to which antibodies bind, we focused our study on this recombinant region of the S-protein rather than the whole molecule of the S-protein.

**Figure 1:**
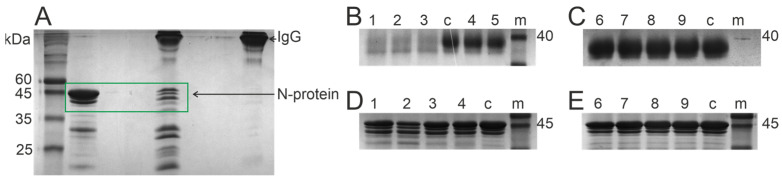
Analysis of N-protein hydrolysis by IgG isolated from the blood of patients who have recovered from COVID-19. The green rectangle shows a fragment of the gel corresponding to the original protein, which was taken into account for calculations (**A**). SDS-PAGE analysis of hydrolysis of the RBD (**B**,**C**) and N-protein (**D**,**E**) with antibodies isolated from the blood of patients who have recovered from COVID-19 (**B**,**D**)—lanes 1–5; depleted antibody subfractions after chromatography on RBD- and N-Sepharose (**C**,**E**)—lanes 6–9. c—reaction mixture which did not contain antibodies, incubated under the same conditions (control). m—protein mass markers RAV11 (Biolabmix, Novosibirsk, Russia).

The IgG concentration in blood plasma is 5–15 mg/mL. Our strategy for determining catalytic activity does not allow for the use of such concentrations in vitro. The biochemical parameters of the reaction depend on the antibody concentration; accordingly, we have to use significantly lower concentrations of antibodies for a longer incubation period. For example, we present an electrophoretic analysis of N-protein hydrolysis by antibodies at a high concentration of 10 mg/mL (Figure 1A). The figure shows a noticeable decrease in the band of the original protein. Unfortunately, the protein used as a substrate is not fully homogeneous, but one can see an increase in the number of products formed. Moreover, in our calculations, we relied on a decrease in the intensity in the band of the original protein, so the impurities did not affect the analysis. For further screening, we used a lower concentration of antibodies and a longer incubation time.

In the first stage, we incubated antibodies from patients who had recovered from COVID-19 with the RBD (Figure 1B) and N-protein (Figure 1D). One can see from Figure 1 that a decrease in the intensity of the band corresponding to the protein was observed after incubation with antibodies (compared to the control samples). Figure 1C,E shows that antibody fractions depleted with corresponding subfractions using affinity chromatography on RBD- and N-Sepharose were not active in the hydrolysis of the relevant proteins. Thus, proteolysis of the RBD and N-protein is associated with the RBD- and N-IgGs, correspondingly. These results are consistent with those in the paper of McConnell et al. [29].

**Figure 2:**
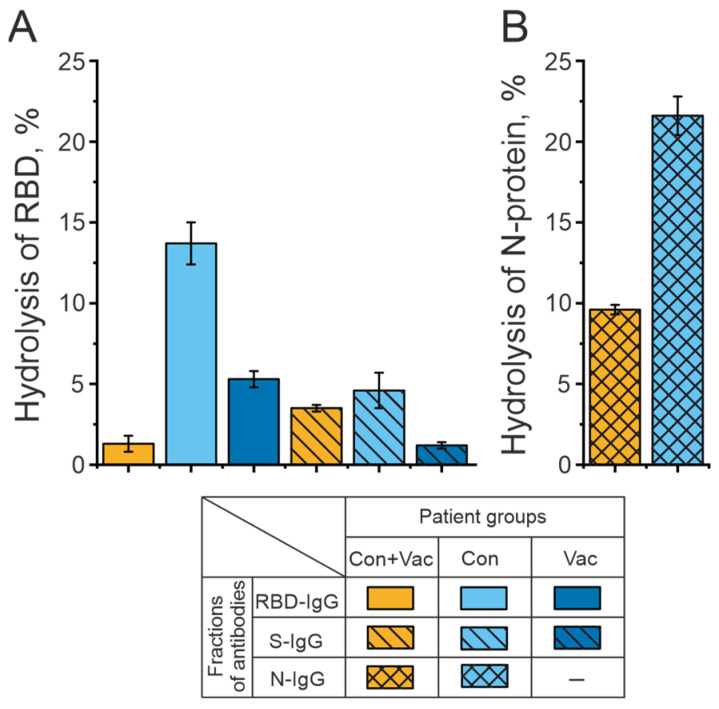
Analysis of RBD (**A**) and N-protein (**B**) hydrolysis by RBD-IgG and S-IgG preparations from COVID-19 convalescents who were subsequently vaccinated with Sputnik V (Con+Vac), COVID-19 convalescents (Con), and patients vaccinated with Sputnik V (Vac). Presented in the figure are the results of a series of three experiments.

In the second stage, the subfractions of RBD-IgG, S-IgG, and N-IgG antibodies were analyzed during the hydrolysis of the RBD and N-proteins of SARS-CoV-2. The relative activity of these antibodies was evaluated through the decrease in the amount of starting proteins in the reaction mixture compared to the control (incubation without IgG). Figure 1B illustrates the analysis of RBD hydrolysis by S-IgG and RBD-IgG preparations of three donor groups. Figure 2B shows the analysis of N-protein hydrolysis by N-IgG preparations from two donor groups. The highest proteolytic activity is characteristic of RBD-IgG and N-IgG from COVID-19 convalescents.

The IgG fractions obtained were examined for cross-reactivity to other viral proteins, including RBD-IgG in N-protein hydrolysis and N-IgG in RBD hydrolysis. The cross-reactivity testing in RBD and N-protein hydrolysis did not provide reliable results for the antibody preparations. These results are consistent with literature data showing that some diseases are associated with antibodies that specifically bind antigens and can hydrolyze them. For example, antibody fractions with an affinity to MPB have been observed in SLE and multiple sclerosis [16,33]. Another example is antibody fractions from HIV-infected patients obtained via affinity chromatography on Sepharose with immobilized HIV-1 integrase. The hydrolysis activity of these antibody fractions is specific to viral integrase and its corresponding oligopeptides, with no effect observed on other proteins under examination [21,34].

Thus, antibodies with affinity to the S- and N-proteins of the SARS-CoV-2 virus prove to hydrolyze only the corresponding substrate and are not cross-reactive toward each other. The highest proteolytic activity was observed in antibody preparations obtained from COVID-19 convalescents. The differences in the hydrolysis activity of viral proteins between the groups can be explained by the individual peculiarities of patients (in the hydrolysis of both proteins) and conformational differences of S-protein of natural origin and that used in vaccines (in the hydrolysis of S-protein). Since the RBD is a region of the Spike protein of SARS-CoV-2, the results obtained in this paper may not be reproduced in the case of hydrolysis of the entire S-protein. However, according to our data and the literature, the RBD is a convenient substrate, and we believe that the results obtained are consistent with what occurs in vivo. Section 2.4 summarizes the specific features of viral protein hydrolysis by specific antibody preparations.

It should be emphasized that the antibodies neutralize the antigen in a few minutes [35,36]; however, the hydrolysis of viral proteins in vitro is a much slower process and, at first glance, does not significantly contribute to the neutralization of the virus. However, this activity may have significant immunological effects in vivo, given the long half-life of antibodies and high titers of circulating antibodies [37,38,39], and the amount of viral proteins relative to antibodies is relatively low. In addition, a low activity level is also characteristic of other proteolytically active antibodies [40,41,42,43].

### 2.3. Intrinsic Catalytic Activity of Antibodies

One of the prerequisites for studying catalytic antibodies is to prove the intrinsic nature of their activity. Modern approaches suggest that attributing catalytic activity directly to antibodies requires a number of criteria to be checked. Such criteria were developed by the group of S. Paul [44] and later extended by the group of G.A. Nevinsky [45]. The following discussion outlines the criteria used to establish the association between proteolytic activity and antibodies found in COVID-19 convalescents or donors or those vaccinated with Sputnik V.

#### 2.3.1. Electrophoretic Homogeneity of IgG Preparations

**Figure 3:**
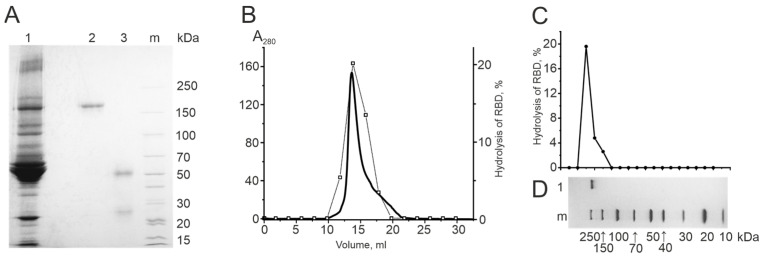
(**A**) 1 indicates the plasma sample of patient 1176, 2 indicates the intact IgG preparation, 3 indicates IgG incubated with 40 mM DTT at 100 °C, and m indicates protein molecular weight markers (cat. #00569095, Thermo Fisher Scientific, Waltham, MA, USA). (**B**) Gel filtration profile of RBD-IgG preparation from COVID-19 convalescents on a Superdex-200 column at pH 2.6. The solid line indicates the optical absorbance of the eluate at λ = 280 nm. The line with squares (□) reflects the relative activity of RBD hydrolysis by RBD-IgG preparation after gel filtration. (**C**) The relative activity of RBD-IgG in RBD hydrolysis after SDS electrophoresis in 4–18% gradient PAGE. (**D**) Electrophoretic analysis of RBD-IgG preparation (Coomassie blue staining of the control lane), where m indicates the protein mass marker and 1 indicates the RBD-IgG sample.

The electrophoretic homogeneity of all IgG preparations was demonstrated via SDS-PAGE analysis, revealing a single band with a molecular mass slightly above 150 kDa in a characteristic region for natural IgG preparations (Figure 3A-2) [31]. To illustrate, Figure 3A-1 shows the electrophoretic separation of blood plasma proteins prior to antibody isolation, with affinity chromatography on Protein G Sepharose allowing for the separation of IgG from other plasma proteins. Figure 3A-3 indicates that incubation of IgG with dithiothreitol (DTT) under heating to 100 °C under conditions of complete disulfide bond reduction results in the formation of only two bands corresponding to ~50 kDa (H-chain) and ~25 kDa (L-chain). No additional bands were detected, indicating that the resulting IgG preparations were electrophoretically homogeneous.

#### 2.3.2. Gel Filtration of Antibodies under “Acid Shock” Conditions

The process of incubating IgG at acidic pH values, followed by gel filtration under identical conditions, facilitates the dissociation and subsequent separation of potential immunoglobulin–antigen complexes. The necessity for such a rigid treatment is due to a wide range of antigen–antibody interaction constants that may reach values of the order of 10^−10^ M [4,46]. The coincidence of the optical density profiles of IgG (A_280_) with the activity profile testifies to the intrinsic catalytic activity of the antibody. Human canonical proteolytic enzymes have a much lower molecular mass than intact IgGs. Therefore, the coincidence of activity and IgGs peaks directly indicates that the IgG samples are not contaminated with canonical proteases.

This criterion was tested by incubating the RBD-IgG preparations from each donor group in 1 M Gly-HCl solution (pH 2.6), followed by gel filtration in the same buffer. A single peak corresponding to IgG was observed on the chromatogram. Figure 3B shows that the optical density profile (A280) obtained from gel filtration coincides with the proteolytic activity profile of IgG.

#### 2.3.3. Determination of the Catalytic Activity of Antibodies in Gel

The current set of criteria includes indicators that unambiguously demonstrate the direct connection between catalytic activity and antibodies, unequivocally excluding any impurities. One such criterion is the method of in situ determination of protein enzymatic activity in the gel. Following the electrophoretic separation of antibodies in SDS-PAGE, the gel was subjected to SDS washing. Subsequently, the gel was divided into small fragments measuring 1.5–3 mm in length, and the catalytic activity of the proteins extracted from these fragments was analyzed. The coincidence of the activity profile with the position of the antibody band on the gel indicates that the catalytic activity belongs to the antibodies. This work tested the eluates obtained from each gel fragment for proteolytic activity. Figure 3C shows that the position of the catalytic activity peak corresponds to the location of the IgG band on the gel lane. The absence of hydrolysis in reactions with eluates from other gel fragments proves that the proteolytic activity belongs to IgG and is not due to protease impurities.

### 2.4. Analysis of the Individual Peculiarities of the Proteolytic Activity of Antibodies in the Patient Groups

**Figure 4:**
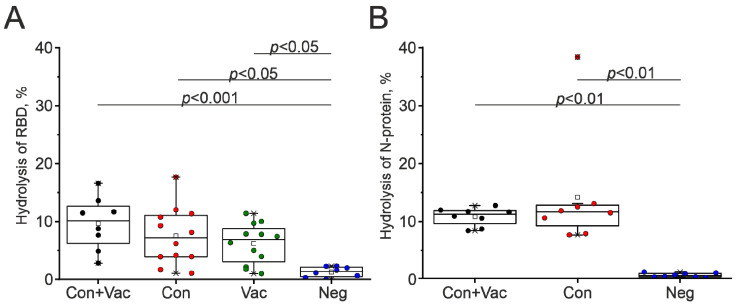
Hydrolysis activity of (**A**) RBD and (**B**) N-protein hydrolysis by the IgG preparations of the selected patient groups: COVID-19 convalescents vaccinated with Sputnik V (Con+Vac, black); COVID-19 convalescents (Con, red); and patients vaccinated with Sputnik V (Vac, green). The data from non-diseased and unvaccinated individuals (Neg, blue) are shown as controls. Presented are the mean values derived from a series of three independent experiments.

In the third stage, we focused on identifying the individual features of patients revealed during the hydrolysis of viral proteins. Blood IgGs of 40 donors from the groups under study were analyzed. According to Figure 4, the antibodies from donors 2145 (16.6%), 2158 (13.6%), and 2134 (11.7%) exhibited the most significant activity in RBD hydrolysis within the Con+Vac group. In the Con group, the antibodies from donors 1176 (17.7%), 1180 (11.4%), and 1171 (10.8%) were the most active in RBD hydrolysis. In the Vac group, the highest proteolytic activity in RBD hydrolysis was observed for the IgG from donors 2041 (11.4%), 2038 (9.7%), and 2021 (10.1%). The antibodies from the blood of the Neg group of donors did not show any detectable proteolytic activity (Figure 4A). The results were statistically significantly different between the Con+Vac and Neg (*p* = 0.0008), Con and Neg (*p* = 0.011), and Vac and Neg (*p* = 0.045) groups.

The activity of N-protein hydrolysis by antibodies from the blood of Con+Vac and Con donors was comparable and amounted to 8.3–12.7% (Figure 4B). An exception was observed in the antibody preparation of patient 2205, which displayed a notably higher N-protein hydrolyzing efficiency of 38.6%. The Kruskal–Wallis one-way ANOVA test revealed statistically significant differences (*p* = 0.0012) between the Con+Vac and Neg groups. As a result, it was found that within each group under study, there were some donors with high activity and some with low activity. We hypothesize that the proteolytic activity toward viral proteins serves as an additional mechanism for the elimination of viral proteins from the body during recovery from COVID-19. The increased proteolytic activity exhibited by antibody preparations may be linked to the persistence of symptoms in some patients, commonly referred to as long COVID [47].

### 2.5. Effect of Divalent Metal Ions on the Catalytic Activity of IgG Preparations

The effect of divalent metal ions on the catalytic activity of antibodies has been described in the literature. The most frequent increase in reaction rate is associated with Ca^2+^, Co^2+^, Mg^2+^, and Mn^2+^ ions [16,48]. For example, it has been shown that the IgG of SLE patients is most active in the presence of Ca^2+^, Mg^2+^, Co^2+^, and Ni^2+^ ions [16]. The increase in the activity of RBD-IgG and S-IgG during the hydrolysis of S-protein oligopeptides has been reported to occur in the presence of Ca^2+^ ions [27].

**Figure 5:**
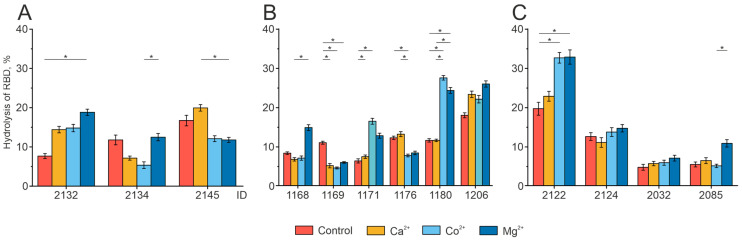
Analysis of RBD hydrolysis by individual antibody preparations in the presence of divalent metal ions. The data are presented for the donors of three groups under study, with (**A**) for COVID-19 convalescents who were then vaccinated (Con+Vac), (**B**) for COVID-19 convalescents (Con), and (**C**) for vaccinated donors (Vac). The preparations incubated without metal ions were used as the control. * *p*-value < 0.05.

The analysis of the effect of these metals on the proteolytic activity of antibodies is presented in Figure 5. The effect of divalent metal ions on the activity of RBD hydrolysis by IgG preparations from different donors is unique. For example, the addition of Mg^2+^ ions to the reaction mixture increased the activity of RBD hydrolysis by antibodies from patients 2132, 1180, and 2085. Co^2+^ and Mg^2+^ ions increased the proteolytic activity of the antibodies of patients 1180 and 2122.

**Figure 6:**
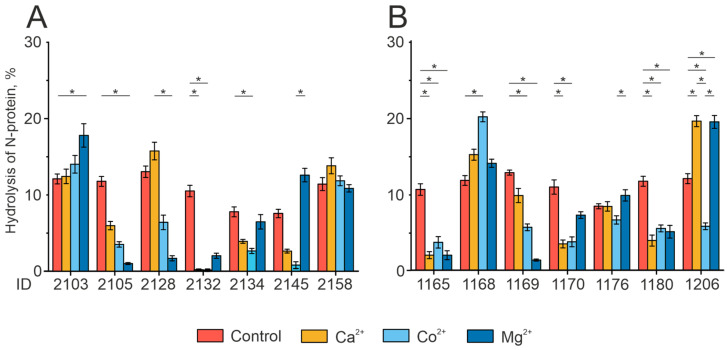
Analysis of N-protein hydrolysis by individual antibody preparations in the presence of divalent metal ions. The data provided include information on two groups under study: (**A**) is for COVID-19 convalescents who were subsequently vaccinated, and (**B**) is for COVID-19 convalescents only. Preparations for the control group were incubated without including metal ions. * *p*-value < 0.05.

The results presented in Figure 6 demonstrate the unique character of the effect of divalent metal ions on the activity of N-protein hydrolysis by IgG preparations from different patients. The addition of Mg^2+^ ions to the reaction mixture increased the activity of RBD hydrolysis by antibodies from patient 2103. Co^2+^ ions produced a positive effect on the proteolytic activity of the antibody preparation from patient 1168. When both Ca^2+^ and Mg^2+^ ions are added, a significant increase was observed in the activity of N-protein hydrolysis by the IgGs from patient 1206, while the addition of Co^2+^ ions, on the other hand, was found to inhibit the reaction.

## 3. Discussion

Catalytic antibodies are part of the immune response to viral infections and autoimmune pathologies. Active interest in catalytic antibodies was shown in the early 1990s and until the mid-2000s; currently, insufficient attention is paid to the study of catalytic antibodies, both in analyzing the immune response to a viral infection and in vaccination. We suggest a positive contribution of catalytic antibodies in the fight against viral infections. First, such antibodies can contribute to the degradation of viral antigens, which contributes to their elimination. Second, it can result from the destabilization of the tertiary and quaternary structure of viral proteins or the hydrolysis of some sites. The destabilization of protein structure, among other factors, might result in conformational changes and heightened accessibility of protein regions crucial for the neutralization of antibodies. A potential synergy between antibody-mediated proteolysis and blocking antibodies may enhance the neutralization process [29]. This synergy between antibody-mediated proteolysis and blocking antibodies is a theoretical mechanism that may enhance the effectiveness of blocking (neutralizing) antibodies. Taken together, we predict that antibody-mediated catalysis is an immunologically significant feature of the humoral immune response that works in concert with the traditional antiviral activity of antibodies. These findings hold great significance in comprehending the physicochemical principles underlying the biological activity of catalytic antibodies.

This study has proven that IgG preparations derived from either COVID-19 convalescents or donors vaccinated with Sputnik V exhibit activity in hydrolyzing the recombinant RBD and N-proteins. Furthermore, it was observed that the antibodies with affinity to the S- and N-proteins of the SARS-CoV-2 virus can hydrolyze only the corresponding substrates and are not cross-reactive. Within each group, donors with higher and lower activity were found. The results are consistent with the literature data. For example, the antibodies from COVID-19 convalescents exhibit proteolytic activity, hydrolyzing the S-protein and two short oligopeptides and contributing to the antiviral function of the antibodies [29]. It should be noted that our study was focused on polyclonal antibodies isolated from the blood plasma of patients. Unfortunately, there are currently no methods for fractionating catalytic antibodies, but our study allows us to evaluate the activity of the antibody pool of patients who have recovered from COVID-19 and vaccinated patients. We also understand that the results of the hydrolysis of the RBD, a region of the SARS-CoV-2 Spike protein, may be reproduced in the case of hydrolysis of the complete trimeric S-protein in vivo but not in full. However, the RBD is an accessible substrate; many scientific publications work specifically with this immunodominant region of the S-protein. Therefore, we believe that the results obtained mainly describe the actual physiological situation that occurs between natural proteolytic antibodies and the receptor-binding domain of the S-protein.

In developing vaccines, considerable attention is paid to generating neutralizing antibodies in response to immunization. However, the immune response is not limited to this type of antibody, and other aspects, such as the formation of catalytic antibodies, should be considered when developing vaccines.

Another fundamental issue is the analysis of antibody-mediated hydrolysis sites. Previously, we conducted a MALDI-TOF analysis of the hydrolysis sites of S-protein-related oligopeptides with antibodies isolated from the blood plasma of patients who have recovered from COVID-19 [28]. It has been shown that catalytic antibodies hydrolyze peptide bonds, but the observed type of proteolysis was not similar to that of proteolytic enzymes, such as trypsin and chymotrypsin. Unique sites for the hydrolysis of oligopeptides were identified, which are characteristic of antibodies only and not classical proteases. From this perspective, we plan to conduct a detailed analysis of the antibody-mediated hydrolysis sites of the RBD and N-proteins.

## 4. Materials and Methods

### 4.1. Donors and Patients

The study was approved by the Local Ethics Committee of the Institute of Chemical Biology and Fundamental Medicine (Protocol Number 21-4 from 15 August 2020), including the written consent of patients and healthy donors to provide their blood for scientific purposes according to the guidelines of the Helsinki ethics committee.

The serology of SARS-CoV-2 IgG against the S-protein and N-protein of SARS-CoV-2 was evaluated using ELISA Antigma G (Generium, Vladimir, Russia) according to the manufacturer’s instructions. The plasma samples of 100 volunteers were collected for this study. All of the patients and donors were categorized into four groups, with each comprising 25 volunteers:

- (Con+Vac group)—COVID-19 convalescents subsequently vaccinated with Sputnik V
- (Con group)—COVID-19 convalescents
- (Vac group)—volunteers vaccinated with Sputnik V
- (Neg group)—a control group of neither infected nor vaccinated donors.

Given that the samples were collected in Novosibirsk between October 2020 and May 2021, these samples are indicative of the antibody response of B-lymphocytes to the Wuhan strain.

### 4.2. Antibody Isolation from Blood Plasma

IgGs were isolated from the blood plasma of patients using affinity chromatography on Protein-G-Sepharose similar to [31]. An equimolar total IgG preparation was fractionated on sorbents with immobilized viral proteins: the RBD, S-protein, and N-protein, resulting in RBD-IgG, S-IgG, and N-IgG fractions, similar to [31].

### 4.3. Gel Filtration under “Acid Shock” Conditions

The IgG preparations (0.8 mL, 1 mg/mL) were incubated in 0.1 mL of 1 M Gly-HCl solution, pH 2.6, for 1 h at 37 °C (conditions for the destruction of strong immune complexes). Subsequently, gel filtration was carried out on a Superdex-200 column (GE Healthcare, Chicago, IL, USA) with a volume of 23 mL. The column was equilibrated with a buffer of 0.1 M Gly-HCl, pH 2.6, containing 150 mM NaCl. Antibodies were eluted with the same buffer at a rate of 0.3 mL/min. The IgG yield from the column was assessed based on the optical density change of the eluate at 280 nm. Following collection, the obtained fractions were neutralized using 1.0 M Tris-HCl at a pH of 8.8. The estimation of their relative activity in the hydrolysis reaction of the RBD or N-protein can be found in Section 4.5.

### 4.4. Analysis of the Proteolytic Activity of Antibodies in the Gel

In order to demonstrate that the antibodies are responsible for the proteolytic activity, we conducted SDS electrophoresis using a 4–15% gradient polyacrylamide gel. Following the electrophoretic separation of proteins, the gel from the control lane was isolated and subjected to staining using Coomassie blue (Sigma, Saint Louis, MO, USA). The gel of the experimental lane underwent a 30 min wash in 4% Triton solution to remove SDS, followed by 4–5 subsequent rinses with water for 10 min each. Next, the gel was sectioned into longitudinal strips, with each strip measuring 3–4 mm in length. These strips were then individually placed in separate tubes and homogenized.

In order to restore protein renaturation and IgG catalytic activity, the gel fragments were subjected to 24 h incubation in TBS buffer at a temperature of 4 °C. The gel was removed via centrifugation (Eppendorf centrifuge, Germany, Hamburg) at 10,000 rpm for 10 min. The catalytic activity of the supernatants (15 µL) was determined in the hydrolysis of the RBD or N-protein, as described in Section 4.4.

### 4.5. Analysis of Proteolytic IgG Activity

The catalytic activity of IgG was assessed by measuring the efficiency of cleavage of the RBD and N-protein. The reaction mixture (20 μL) contained 20 mM Tris-HCl, pH 7.5, IgG preparation (0.1 mg/mL), and the RBD or N-protein (0.3 mg/mL). The mixture was incubated for 48 h at 37 °C. The efficiency of RBD or N-protein hydrolysis was analyzed by decreasing the band intensity of the intact RBD or N-protein after the separation of reaction products via SDS-electrophoresis. For this purpose, the staining intensity of RBD or N-protein and its hydrolysis products were compared with the control lane proteins using ImageQuant 5.2 software (Molecular Dynamics).

### 4.6. Investigation of the Effect of Various Metal Ions on Antibody Catalytic Activity

In order to remove endogenous metal ions from the IgG preparations, their dialysis was performed against a buffer solution containing 20 mM Tris-HCl, pH 7.5, 0.1 M NaCl, and 0.1 M EDTA. This was followed by a second dialysis step using the same deionized buffer but without EDTA. The final 20 μL reaction mixture contained 20 mM Tris-HCl, pH 7.5, 0.4 mg/mL IgG, 0.3 mg/mL RBD, and 2 mM divalent metal chloride (Co^2+^, Ca^2+^, Mg^2+^). The reaction mixtures containing no metal ions were used as controls. IgG samples were incubated with the reaction mixture for 48 h at 37° C. The efficiency of RBD hydrolysis by IgG preparations in the presence of metals was established by measuring the reduction in color intensity of the protein band on a polyacrylamide gel compared to the protein band incubated without metal ions, as described in Section 4.5. The impact of divalent metal ions on the hydrolysis activity of the N-protein during reactions with IgGs was assessed in a similar manner.

### 4.7. Statistical Analysis

The significance of differences between groups of IgG preparations was analyzed using the Kruskal–Wallis one-way ANOVA test for non-parametric samples in STATISTICA 10 software. A *p*-value of less than 0.05 was considered to be statistically significant.

## 5. Conclusions

In this work, we analyzed antibodies formed after COVID-19 infection and/or vaccination in the hydrolysis of SARS-CoV-2 proteins. Our findings demonstrate that IgGs from individuals who recovered from COVID-19 or received the Sputnik V vaccine exhibit hydrolytic activity toward the recombinant RBD and N-protein. Furthermore, this activity is exclusively observed in antibodies that target these specific proteins. Consequently, proteolytic antibodies can specifically hydrolyze viral proteins and have a significant impact on the pathogenesis of COVID-19.

Antibody-mediated proteolysis of the S-protein of SARS-CoV-2 is associated with virus neutralization, indicating that immunoglobulins have an active role in controlling viral infections. For example, catalytic antibodies that recognize distinct epitopes of the S-protein can directly affect neutralization by destabilizing the tertiary and quaternary structure of the S-protein. However, these antibodies are not limited to conformational screening of important epitopes, such as the RBD. It cannot be excluded that proteolytic antibodies work in combination with blocking antibodies to enhance the neutralization process.

Therefore, proteolytic catalysis mediated by antibodies represents an immunologically significant characteristic of the humoral immune response in COVID-19 infection, functioning in coordination with the antibody’s conventional antiviral function. These findings hold significant implications for comprehending the biological efficacy of naturally produced antibodies following infection and/or vaccination.

## Data Availability

Empirical data that do not relate to the personal data of donors can be provided by Anna Timofeeva upon request.
